# Normothermic machine perfusion in liver transplantation: a bibliometric analysis of the top 100 most cited articles

**DOI:** 10.1097/MS9.0000000000003222

**Published:** 2025-03-28

**Authors:** Hiba Thasleem, Muhammad Ahmad Nadeem, Hamza Ashraf, Ghazal Ishaque, Mahinn Saadi, Mansoor Ahmed, Mian Zahid Jan Kakakhel, Abdul Rafeh Awan, Aalaa Saleh, Amir H Sohail

**Affiliations:** a Fatima Jinnah Medical University, Lahore, Pakistan; b Digestive Diseases and Surgery Institute, Cleveland Clinic, Cleveland, Ohio, USA; c Allama Iqbal Medical College, Lahore, Pakistan; d Shaheed Mohtarma Benazir Bhutto Medical College, Karachi, Pakistan; e Dow University of Health Sciences, Karachi, Pakistan; f Liaquat University of Medical and Health Sciences, Jamshoro, Pakistan; g Rehman Medical College, Peshawar, Pakistan; h Nishtar Medical University, Multan, Pakistan; i Faculty of Medicine, Lebanese University, Beirut, Lebanon; j Department of Surgery, University of New Mexico, Albuquerque, New Mexico, USA

**Keywords:** liver grafting, liver transplantation, normothermic machine perfusion, normothermic perfusion and hepatic grafting, normothermic preservation

## Abstract

**Background:**

Normothermic machine perfusion (NMP) has increased substantially in the recent decade, being a vital tool in further organ preservation and reducing ischemia-reperfusion injury. The purpose of this study was to objectively conduct a bibliometric analysis of the top 100 cited articles to understand the evolution of NMP in liver transplantation.

**Methods:**

Scopus was selected as our primary database. We explored the database to extract relevant articles, which were then ranked numerically by the number of citations. A list of the top 100 articles was created in descending order, and each article was further analyzed to identify trends and characteristics. A list of the top 10 review articles was also prepared.

**Results:**

The top 100 studies were cited a total of 6136 times from 2013 and 2023, with the most cited articles published in 2018. The total number of citations per article ranged from 7 to 787, with a median of 397 citations. The articles originated from 13 different countries, with the United Kingdom having the most articles (*n* = 26), followed by the Netherlands (*n* = 17) and the United States (*n* = 17). *Liver Transplantation* (*n* = 21)*, Transplantation* (*n* = 10)*, American Journal of Transplantation* (*n* = 10)*, and Annals of Surgery* (*n* = 6) contributed to nearly half of the articles.

**Conclusion:**

Research on NMP is rapidly growing and encompasses a variety of countries and institutions. Our analysis provides insight into the evolution of normothermic machine perfusion in liver transplantation, with the hope that this article may serve as a reference to aid healthcare professionals in efficiently assessing consensus, trends, and needs within the field.

## Introduction

The growing gap between the supply and demand for organ donations has led to the acceptance of organs from extended criteria donors. This principle also applies to liver transplantation^[1]^. Effective preservation techniques were needed to ensure graft survival from procurement to implantation to achieve successful transplantation with livers from these sources^[2]^. Thus, work began to find a suitable technique for preserving donor livers to ensure a good graft survival outcome. Keeping donor livers in static cold storage in a preservation solution from the time of procurement to the time of implantation is not ideal for extended criteria donors as these grafts are more prone to ischemia-reperfusion injury.^[3]^ While the low-temperature environment (0–4°C) slows organ metabolism^[4]^, it does not completely halt it. Consequently, the absence of metabolic substrates and the accumulation of metabolites can still lead to donor liver damage^[5,6]^. As a result, CS alone may not be sufficient to maintain the quality of marginal donor livers. In the past decade, transplant surgeons have published extensively on donor liver preservation techniques. One successful method that has emerged to provide an almost normal physiological environment for the organs ex-vivo is normothermic machine perfusion.

**HIGHLIGHTS**

- There was a rapid increase in citation activity for normothermic machine perfusion (NMP) articles from 2015 to 2020, reflecting growing interest and advancements in liver transplantation research.
- The United Kingdom, the Netherlands, and the United States led in high-impact NMP publications, with University Hospitals Birmingham and University Medical Center Groningen as top contributing institutions.
- The study highlighted significant gender (85% male first authors, 95% male senior authors) and racial disparities, emphasizing the need for diversity in NMP research.

Normothermic machine perfusion (NMP) has emerged as a promising organ preservation technique, helping reduce ischemia-reperfusion injury and improve outcomes for extended criteria donor liver grafts.^[7-10]^ Additionally, NMP allows graft viability and function to be assessed before transplantation.^[11,12]^ NMP operates on the principle that organs can be preserved at physiological temperatures outside the body while maintaining metabolic functions. Perfusion devices vary in how they deliver blood flow to the liver, but to meet the metabolic demands of the graft, ex-situ perfusion solutions require an oxygen carrier to supply the necessary oxygen.^[13]^ While many journals offer statistics in their publications, we found no comprehensive bibliometric analysis of NMP in liver transplantation. Thus, we have conducted one on normothermic machine perfusion in liver transplantation. Bibliometric analysis evaluates published literature’s citation frequency trends and assesses its impact within a specific field. Through citation analysis, it identifies the most productive authors, countries, and journals in that area of interest. This process highlights notable works and helps institutions allocate their limited resources toward authors and organizations that produce high-quality research. Additionally, bibliometric analysis reveals trends in funding for published literature and disparities related to gender and race among authors.

## Methods

We selected Scopus (http://www.scopus.com) as our primary database due to its more reliable citation counts and broader journal indexing, offering approximately 20% greater coverage than the Web of Science.^[14]^ In contrast, PubMed (MEDLINE) and Embase do not track citation counts, while Google Scholar is considered unreliable for bibliometric analyses.^[15]^ Two reviewers, A.N and M.S, independently conducted searches in Scopus in September 2024. The search was limited to original articles and studies involving non-human subjects, and articles in languages other than English were excluded. No specific time frame was applied to the inclusion or exclusion criteria, and all journals within the database were considered.

**Figure 1.**
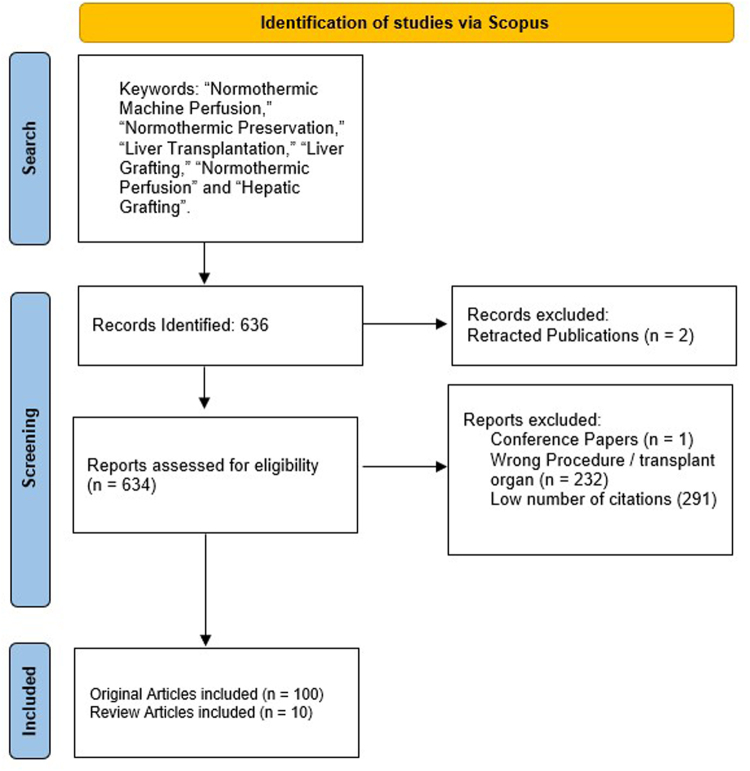
The article selection process using Scopus database.

The primary search keywords included “Normothermic Machine Perfusion,” “Normothermic Preservation,” “Liver Transplantation,” and “Liver Grafting,” with expanded terms such as “Normothermic Perfusion” and “Hepatic Grafting” included as well. These keywords were searched within the titles, abstracts, keywords, and full text. Only studies primarily focused on Normothermic Perfusion in liver transplantation were included. A total of 636 articles were retrieved and imported into an Excel file. If an abstract was unavailable in Scopus, it was sourced from alternative platforms for relevance assessment. The retrieved articles were then organized using the “cited by” feature, and the top 100 most-cited articles were compiled into a preliminary list and then reviewed to exclude irrelevant articles. The selection process for the articles has been detailed in Fig. 1.

For each article in the final list, we recorded the total citation count, year of publication, journal name, Impact Factor (IF), number of authors and their H-index, country of origin, type of study, and funding sources. Citations per year were calculated for all articles. Journal Impact Factors were obtained from the Journal Citation Report. ResearchGate profiles and Google Scholar were utilized to determine the authors’ countries of origin. The current affiliation was used for simplicity for authors affiliated with multiple countries. The gender of the first, second, third, and last authors was determined by reviewing their institutional website photos or noting pronouns used in their profiles. In cases where an article had a single author, that individual was considered the senior author.

The normality of the quantitative data was assessed using the Shapiro-Wilk test, and statistical tests were selected accordingly based on whether the data followed a normal or skewed distribution. IBM SPSS Statistics (v20.0 International Business Machines, IL, USA) was used to apply the Pearson correlation coefficient test to evaluate the association between IF of a journal and the total number of citations it garnered and its association with the number of articles included in the list. Mann-Whitney U test was applied to investigate the association of funding, conflict of interests, and gender with the number of citations. χ2 test was used to determine if there was an association between the sex of the first and senior authors. A *P*-value <0.05 was considered significant in all cases. In addition, co-citation analysis was performed using “Vos Viewer” (version 1.6.6) for better network visualization among authors.

## Results

### Citation count, citations per year, and citation trend

Table 1 shows the 100 most cited articles on Normothermic Machine Perfusion, with each article’s total citations and citations per year. The number of citations of these 100 articles ranged from 7 to 787, with a median of 24.5 (interquartile range of 69). The sum of the citations is 6136. Approximately 18% of the total citations were self-citations. The citations per year ranged from 1.33 to 131.1, with a mean of 14.4 and a median of 11 (interquartile range of 11.77)

**Table 1:** The top 100 most cited articles

Rank	Articles	Citations	Year	Citations/year
**1**	Nasralla D.; Coussios C.C.; Mergental H.; Akhtar M.Z.; Butler A.J.; Ceresa C.D.L.; Chiocchia V.; Dutton S.J.; García-Valdecasas J.C.; Heaton N.; Imber C.; Jassem W.; Jochmans I.; Karani J.; Knight S.R.; Kocabayoglu P.; Malagò M.; Mirza D.; Morris P.J.; Pallan A.; Paul A.; Pavel M.; Perera M.T.P.R.; Pirenne J.; Ravikumar R.; Russell L.; Upponi S.; Watson C.J.E.; Weissenbacher A.; Ploeg R.J.; Friend P.J., A randomized trial of normothermic preservation in liver transplantation, Nature, 7703, 2018.	787	2018	131.17
**2**	Ravikumar R.; Jassem W.; Mergental H.; Heaton N.; Mirza D.; Perera M.T.P.R.; Quaglia A.; Holroyd D.; Vogel T.; Coussios C.C.; Friend P.J., Liver transplantation after ex vivo normothermic machine preservation: a phase 1 (First-in-Man) clinical trial, American Journal of Transplantation, 6, 2016.	374	2016	46.75
**3**	Mergental H.; Laing R.W.; Kirkham A.J.; Perera M.T.P.R.; Boteon Y.L.; Attard J.; Barton D.; Curbishley S.; Wilkhu M.; Neil D.A.H.; Hübscher S.G.; Muiesan P.; Isaac J.R.; Roberts K.J.; Abradelo M.; Schlegel A.; Ferguson J.; Cilliers H.; Bion J.; Adams D.H.; Morris C.; Friend P.J.; Yap C.; Afford S.C.; Mirza D.F., Transplantation of discarded livers following viability testing with normothermic machine perfusion, Nature Communications, 1, 2020.	269	2020	67.25
**4**	Mergental H.; Perera M.T.P.R.; Laing R.W.; Muiesan P.; Isaac J.R.; Smith A.; Stephenson B.T.F.; Cilliers H.; Neil D.A.H.; Hübscher S.G.; Afford S.C.; Mirza D.F., Transplantation of declined liver allografts following normothermic ex-situ evaluation, American Journal of Transplantation, 11, 2016.	255	2016	31.87
**5**	Eshmuminov D.; Becker D.; Bautista Borrego L.; Hefti M.; Schuler M.J.; Hagedorn C.; Muller X.; Mueller M.; Onder C.; Graf R.; Weber A.; Dutkowski P.; Rudolf von Rohr P.; Clavien P.A., An integrated perfusion machine preserves injured human livers for 1 week, Nature Biotechnology, 2, 2020.	235	2020	58.75
**6**	Op Den Dries S.; Karimian N.; Sutton M.E.; Westerkamp A.C.; Nijsten M.W.N.; Gouw A.S.H.; Wiersema-Buist J.; Lisman T.; Leuvenink H.G.D.; Porte R.J., Ex vivo normothermic machine perfusion and viability testing of discarded human donor livers, American Journal of Transplantation, 5, 2013.	234	2013	21.27
**7**	Markmann J.F.; Abouljoud M.S.; Ghobrial R.M.; Bhati C.S.; Pelletier S.J.; Lu A.D.; Ottmann S.; Klair T.; Eymard C.; Roll G.R.; Magliocca J.; Pruett T.L.; Reyes J.; Black S.M.; Marsh C.L.; Schnickel G.; Kinkhabwala M.; Florman S.S.; Merani S.; Demetris A.J.; Kimura S.; Rizzari M.; Saharia A.; Levy M.; Agarwal A.; Cigarroa F.G.; Eason J.D.; Syed S.; Washburn W.K.; Parekh J.; Moon J.; Maskin A.; Yeh H.; Vagefi P.A.; MacConmara M.P., Impact of portable normothermic blood-based machine perfusion on outcomes of liver transplant: The OCS Liver PROTECT Randomized Clinical Trial, JAMA Surgery, 3, 2022.	172	2022	86.00
**8**	Bral M.; Gala-Lopez B.; Bigam D.; Kneteman N.; Malcolm A.; Livingstone S.; Andres A.; Emamaullee J.; Russell L.; Coussios C.; West L.J.; Friend P.J.; Shapiro A.M.J., Preliminary single-center Canadian experience of human normothermic ex vivo liver perfusion: results of a clinical trial, American Journal of Transplantation, 4, 2017.	168	2017	24.00
**9**	Van Leeuwen O.B.; De Vries Y.; Fujiyoshi M.; Nijsten M.W.N.; Ubbink R.; Pelgrim G.J.; Werner M.J.M.; Reyntjens K.M.E.M.; Van Den Berg A.P.; De Boer M.T.; De Kleine R.H.J.; Lisman T.; De Meijer V.E.; Porte R.J., Transplantation of high-risk donor livers after ex situ resuscitation and assessment using combined hypo- and normothermic machine perfusion: a prospective clinical trial. Annals of Surgery, 2019.	161	2019	32.20
**10**	Sutton M.E.; Op Den Dries S.; Karimian N.; Weeder P.D.; De Boer M.T.; Wiersema-Buist J.; Gouw A.S.H.; Leuvenink H.G.D.; Lisman T.; Porte R.J., Criteria for viability assessment of discarded human donor livers during ex vivo normothermic machine perfusion, PLoS ONE, 11, 2014.	152	2014	15.20
**11**	Matton A.P.M.; De Vries Y.; Burlage L.C.; Van Rijn R.; Fujiyoshi M.; De Meijer V.E.; De Boer M.T.; De Kleine R.H.J.; Verkade H.J.; Gouw A.S.H.; Lisman T.; Porte R.J., Biliary bicarbonate, pH, and glucose are suitable biomarkers of biliary viability during ex situ normothermic machine perfusion of human donor livers, Transplantation, 7, 2019.	126	2019	25.20
**12**	de Vries Y.; Matton A.P.M.; Nijsten M.W.N.; Werner M.J.M.; van den Berg A.P.; de Boer M.T.; Buis C.I.; Fujiyoshi M.; de Kleine R.H.J.; van Leeuwen O.B.; Meyer P.; van den Heuvel M.C.; de Meijer V.E.; Porte R.J., Pretransplant sequential hypo- and normothermic machine perfusion of suboptimal livers donated after circulatory death using a hemoglobin-based oxygen carrier perfusion solution, American Journal of Transplantation, 4, 2019.	118	2019	23.60
**13**	He X.; Guo Z.; Zhao Q.; Ju W.; Wang D.; Wu L.; Yang L.; Ji F.; Tang Y.; Zhang Z.; Huang S.; Wang L.; Zhu Z.; Liu K.; Zhu Y.; Gao Y.; Xiong W.; Han M.; Liao B.; Chen M.; Ma Y.; Zhu X.; Huang W.; Cai C.; Guan X.; Li X.C.; Huang J., The first case of ischemia-free organ transplantation in humans: a proof of concept, American Journal of Transplantation, 3, 2018.	109	2018	18.16
**14**	Jassem W.; Xystrakis E.; Ghnewa Y.G.; Yuksel M.; Pop O.; Martinez-Llordella M.; Jabri Y.; Huang X.; Lozano J.J.; Quaglia A.; Sanchez-Fueyo A.; Coussios C.C.; Rela M.; Friend P.; Heaton N.; Ma Y., Normothermic machine perfusion (NMP) inhibits proinflammatory responses in the liver and promotes regeneration, Hepatology, 2, 2019.	108	2019	21.60
**15**	Boteon Y.L.; Attard J.; Boteon A.P.C.S.; Wallace L.; Reynolds G.; Hubscher S.; Mirza D.F.; Mergental H.; Bhogal R.H.; Afford S.C., Manipulation of lipid metabolism during normothermic machine perfusion: effect of defatting therapies on donor liver functional recovery, Liver Transplantation, 7, 2019.	97	2019	19.40
**16**	Perera T.; Mergental H.; Stephenson B.; Roll G.R.; Cilliers H.; Liang R.; Angelico R.; Hubscher S.; Neil D.A.; Reynolds G.; Isaac J.; Adams D.A.; Afford S.; Mirza D.F.; Muiesan P., First human liver transplantation using a marginal allograft resuscitated by normothermic machine perfusion, Liver Transplantation, 1, 2016.	95	2016	11.87
**17**	Westerkamp A.C.; Karimian N.; Matton A.P.M.; Mahboub P.; Van Rijn R.; Wiersema-Buist J.; De Boer M.T.; Leuvenink H.G.D.; Gouw A.S.H.; Lisman T.; Porte R.J., Oxygenated hypothermic machine perfusion after static cold storage improves hepatobiliary function of extended criteria donor livers, Transplantation, 4, 2016.	92	2016	11.50
**18**	Ghinolfi D.; Rreka E.; De Tata V.; Franzini M.; Pezzati D.; Fierabracci V.; Masini M.; Cacciatoinsilla A.; Bindi M.L.; Marselli L.; Mazzotti V.; Morganti R.; Marchetti P.; Biancofiore G.; Campani D.; Paolicchi A.; De Simone P., Pilot, open, randomized, prospective trial for normothermic machine perfusion evaluation in liver transplantation from older donors, Liver Transplantation, 3, 2019.	91	2019	18.20
**19**	Laing R.W.; Mergental H.; Yap C.; Kirkham A.; Whilku M.; Barton D.; Curbishley S.; Boteon Y.L.; Neil D.A.; Hübscher S.G.; Thamara Perera M.P.R.; Muiesan P.; Isaac J.; Roberts K.J.; Cilliers H.; Afford S.C.; Mirza D.F., Viability testing and transplantation of marginal livers (VITTAL) using normothermic machine perfusion: Study protocol for an open-label, non-randomised, prospective, single-arm trial, BMJ Open, 11, 2017.	90	2017	12.85
**20**	Mergental H.; Stephenson B.T.F.; Laing R.W.; Kirkham A.J.; Neil D.A.H.; Wallace L.L.; Boteon Y.L.; Widmer J.; Bhogal R.H.; Perera M.T.P.R.; Smith A.; Reynolds G.M.; Yap C.; Hübscher S.G.; Mirza D.F.; Afford S.C., Development of clinical criteria for functional assessment to predict primary nonfunction of high-risk livers using normothermic machine perfusion, Liver Transplantation, 10, 2018.	88	2018	14.66
**21**	Watson C.J.E.; Kosmoliaptsis V.; Randle L.V.; Russell N.K.; Griffiths W.J.H.; Davies S.; Mergental H.; Butler A.J., Preimplant normothermic liver perfusion of a suboptimal liver donated after circulatory death, American Journal of Transplantation, 1, 2016.	88	2016	11.00
**22**	Clavien P.-A.; Dutkowski P.; Mueller M.; Eshmuminov D.; Bautista Borrego L.; Weber A.; Muellhaupt B.; Sousa Da Silva R.X.; Burg B.R.; Rudolf von Rohr P.; Schuler M.J.; Becker D.; Hefti M.; Tibbitt M.W., Development of clinical criteria for functional assessment to predict primary nonfunction of high-risk livers using normothermic machine perfusion, Liver Transplantation, 10, 2018.	88	2018	14.66
**23**	Boteon Y.L.; Laing R.W.; Schlegel A.; Wallace L.; Smith A.; Attard J.; Bhogal R.H.; Neil D.A.H.; Hübscher S.; Perera M.T.P.R.; Mirza D.F.; Afford S.C.; Mergental H., Combined hypothermic and normothermic machine perfusion improves functional recovery of extended criteria donor livers, Liver Transplantation, 12, 2018.	84	2018	14.00
**24**	Vogel T.; Brockmann J.G.; Quaglia A.; Morovat A.; Jassem W.; Heaton N.D.; Coussios C.C.; Friend P.J., The 24-hour normothermic machine perfusion of discarded human liver grafts, Liver Transplantation, 2, 2017.	83	2017	11.85
**25**	Matton A.P.M.; Burlage L.C.; van Rijn R.; de Vries Y.; Karangwa S.A.; Nijsten M.W.; Gouw A.S.H.; Wiersema-Buist J.; Adelmeijer J.; Westerkamp A.C.; Lisman T.; Porte R.J., Normothermic machine perfusion of donor livers without the need for human blood products, Liver Transplantation, 4, 2018.	81	2018	13.50
**26**	Cardini B.; Oberhuber R.; Fodor M.; Hautz T.; Margreiter C.; Resch T.; Scheidl S.; Maglione M.; Bösmüller C.; Mair H.; Frank M.; Augustin F.; Griesmacher A.; Schennach H.; Martini J.; Breitkopf R.; Eschertzhuber S.; Pajk W.; Obwegeser A.; Tilg H.; Watson C.; Öfner D.; Weissenbacher A.; Schneeberger S., Clinical implementation of prolonged liver preservation and monitoring through normothermic machine perfusion in liver transplantation, Transplantation, 9, 2020.	81	2020	20.25
**27**	De Carlis R.; Di Sandro S.; Lauterio A.; Ferla F.; Dell’Acqua A.; Zanierato M.; De Carlis L., Successful donation after cardiac death liver transplants with prolonged warm ischemia time using normothermic regional perfusion, Liver Transplantation, 2, 2017.	78	2017	11.14
**28**	Clavien P.-A.; Dutkowski P.; Mueller M.; Eshmuminov D.; Bautista Borrego L.; Weber A.; Muellhaupt B.; Sousa Da Silva R.X.; Burg B.R.; Rudolf von Rohr P.; Schuler M.J.; Becker D.; Hefti M.; Tibbitt M.W., Transplantation of a human liver following 3 days of ex situ normothermic preservation, Nature Biotechnology, 11, 2022.	74	2022	37.00
**29**	Ceresa C.D.L.; Nasralla D.; Watson C.J.E.; Butler A.J.; Coussios C.C.; Crick K.; Hodson L.; Imber C.; Jassem W.; Knight S.R.; Mergental H.; Ploeg R.J.; Pollok J.M.; Quaglia A.; Shapiro A.M.J.; Weissenbacher A.; Friend P.J., Transient cold storage prior to normothermic liver perfusion may facilitate adoption of a novel technology, Liver Transplantation, 10, 2019.	67	2019	13.40
**30**	van Leeuwen O.B.; Bodewes S.B.; Lantinga V.A.; Haring M.P.D.; Thorne A.M.; Brüggenwirth I.M.A.; van den Berg A.P.; de Boer M.T.; de Jong I.E.M.; de Kleine R.H.J.; Lascaris B.; Nijsten M.W.N.; Reyntjens K.M.E.M.; de Meijer V.E.; Porte R.J., Sequential hypothermic and normothermic machine perfusion enables safe transplantation of high-risk donor livers, American Journal of Transplantation, 6, 2022.	63	2022	31.50
**31**	De Carlis R.; Schlegel A.; Frassoni S.; Olivieri T.; Ravaioli M.; Camagni S.; Patrono D.; Bassi D.; Pagano D.; Di Sandro S.; Lauterio A.; Bagnardi V.; Gruttadauria S.; Cillo U.; Romagnoli R.; Colledan M.; Cescon M.; Di Benedetto F.; Muiesan P.; De Carlis L., How to preserve liver grafts from circulatory death with long warm ischemia? A retrospective Italian cohort study with normothermic regional perfusion and hypothermic oxygenated perfusion, Transplantation, 11, 2021.	63	2021	21.00
**32**	Gaurav R.; Butler A.J.; Kosmoliaptsis V.; Mumford L.; Fear C.; Swift L.; Fedotovs A.; Upponi S.; Khwaja S.; Richards J.; Allison M.; Watson C.J.E., Liver transplantation outcomes from controlled circulatory death donors: SCS vs in situ NRP vs ex situ NMP, Annals of Surgery, 6, 2022.	62	2022	31.00
**33**	Angelico R.; Perera M.P.R.T.; Ravikumar R.; Holroyd D.; Coussios C.; Mergental H.; Isaac J.R.; Iqbal A.; Cilliers H.; Muiesan P.; Friend P.J.; Mirza D.F., Normothermic machine perfusion of deceased donor liver grafts is associated with improved postreperfusion hemodynamics, Transplantation Direct, 9, 2016.	55	2016	6.87
**34**	Bral M.; Dajani K.; Leon Izquierdo D.; Bigam D.; Kneteman N.; Ceresa C.D.L.; Friend P.J.; Shapiro A.M.J., A back-to-base experience of human normothermic ex situ liver perfusion: does the chill kill?, Liver Transplantation, 6, 2019.	55	2019	11.00
**35**	Liu Q.; Nassar A.; Buccini L.; Iuppa G.; Soliman B.; Pezzati D.; Hassan A.; Blum M.; Baldwin W.; Bennett A.; Chavin K.; Okamoto T.; Uso T.D.; Fung J.; Abu-Elmagd K.; Miller C.; Quintini C., Lipid metabolism and functional assessment of discarded human livers with steatosis undergoing 24 hours of normothermic machine perfusion, Liver Transplantation, 2, 2018.	53	2018	8.833
**36**	Macconmara M.; Hanish S.I.; Hwang C.S.; De Gregorio L.; Desai D.M.; Feizpour C.A.; Tanriover B.; Markmann J.F.; Zeh H.; Vagefi P.A., Making every liver count: increased transplant yield of donor livers through normothermic machine perfusion, Annals of Surgery, 3, 2020.	51	2020	12.75
**37**	Ghinolfi D.; Dondossola D.; Rreka E.; Lonati C.; Pezzati D.; Cacciatoinsilla A.; Kersik A.; Lazzeri C.; Zanella A.; Peris A.; Maggioni M.; Biancofiore G.; Reggiani P.; Morganti R.; De Simone P.; Rossi G., Sequential use of normothermic regional and ex situ machine perfusion in donation after circulatory death liver transplant, Liver Transplantation, 3, 2021.	48	2021	16.00
**38**	Wang L.; Thompson E.; Bates L.; Pither T.L.; Hosgood S.A.; Nicholson M.L.; Watson C.J.E.; Wilson C.; Fisher A.J.; Ali S.; Dark J.H., Flavin mononucleotide as a biomarker of organ quality—a pilot study, Transplantation Direct, 9, 2020.	45	2020	11.25
**39**	Muller X.; Mohkam K.; Mueller M.; Schlegel A.; Dondero F.; Sepulveda A.; Savier E.; Scatton O.; Bucur P.; Salame E.; Jeddou H.; Sulpice L.; Pittau G.; Allard M.A.; Mabrut J.Y.; Dutkowski P.; Clavien P.A.; Lesurtel M., Hypothermic oxygenated perfusion versus normothermic regional perfusion in liver transplantation from controlled donation after circulatory death: first international comparative study, Annals of Surgery, 5, 2020.	44	2020	11.00
**40**	Laing R.W.; Stubblefield S.; Wallace L.; Roobrouck V.D.; Bhogal R.H.; Schlegel A.; Boteon Y.L.; Reynolds G.M.; Ting A.E.; Mirza D.F.; Newsome P.N.; Mergental H.; Afford S.C., The delivery of multipotent adult progenitor cells to extended criteria human donor livers using normothermic machine perfusion, Frontiers in Immunology, 2020.	41	2020	10.25
**41**	Fodor M.; Cardini B.; Peter W.; Weissenbacher A.; Oberhuber R.; Hautz T.; Otarashvili G.; Margreiter C.; Maglione M.; Resch T.; Krendl F.; Meszaros A.T.; Bogensperger C.; Gasteiger S.; Messner F.; Henninger B.; Zoller H.; Tilg H.; Öfner D.; Schneeberger S., Static cold storage compared with normothermic machine perfusion of the liver and effect on ischaemic-type biliary lesions after transplantation: a propensity score-matched study, British Journal of Surgery, 9, 2021.	41	2021	13.66
**42**	Agopian V.G.; Markovic D.; Klintmalm G.B.; Saracino G.; Chapman W.C.; Vachharajani N.; Florman S.S.; Tabrizian P.; Haydel B.; Nasralla D.; Friend P.J.; Boteon Y.L.; Ploeg R.; Harlander-Locke M.P.; Xia V.; DiNorcia J.; Kaldas F.M.; Yersiz H.; Farmer D.G.; Busuttil R.W., Multicenter validation of the liver graft assessment following transplantation (L-GrAFT) score for assessment of early allograft dysfunction, Journal of Hepatology, 4, 2021.	37	2021	12.33
**43**	Burlage L.C.; Karimian N.; Westerkamp A.C.; Visser N.; Matton A.P.M.; van Rijn R.; Adelmeijer J.; Wiersema-Buist J.; Gouw A.S.H.; Lisman T.; Porte R.J., Oxygenated hypothermic machine perfusion after static cold storage improves endothelial function of extended criteria donor livers, HPB, 6, 2017.	37	2017	5.28
**44**	Reiling J.; Butler N.; Simpson A.; Hodgkinson P.; Campbell C.; Lockwood D.; Bridle K.; Santrampurwala N.; Britton L.; Crawford D.; Dejong C.H.C.; Fawcett J., Assessment and transplantation of orphan donor livers: a back-to-base approach to normothermic machine perfusion, Liver Transplantation, 12, 2020.	36	2020	9.00
**45**	De Vries Y.; Berendsen T.A.; Fujiyoshi M.; Van Den Berg A.P.; Blokzijl H.; De Boer M.T.; Van Der Heide F.; De Kleine R.H.J.; Van Leeuwen O.B.; Matton A.P.M.; Werner M.J.M.; Lisman T.; De Meijer V.E.; Porte R., Transplantation of high-risk donor livers after resuscitation and viability assessment using a combined protocol of oxygenated hypothermic, rewarming and normothermic machine perfusion: Study protocol for a prospective, single-arm study (DHOPE-COR-NMP trial), BMJ Open, 8, 2019.	35	2019	7.00
**46**	Quintini C.; Del Prete L.; Simioni A.; Del Angel L.; Diago Uso T.; D’Amico G.; Hashimoto K.; Aucejo F.; Fujiki M.; Eghtesad B.; Sasaki K.; Kwon C.H.D.; Cywinski J.; Bennett A.; Bilancini M.; Miller C.; Liu Q., Transplantation of declined livers after normothermic perfusion, Surgery (United States), 3, 2022.	34	2022	17.00
**47**	Liu Q.; Hassan A.; Pezzati D.; Soliman B.; Lomaglio L.; Grady P.; Del Angel Diaz L.; Simioni A.; Maikhor S.; Etterling J.; D’Amico G.; Iuppa G.; Diago Uso T.; Hashimoto K.; Aucejo F.; Fujiki M.; Eghtesad B.; Sasaki K.; Kwon C.H.D.; Cywinski J.; Irefin S.; Bennett A.; Baldwin W.; Miller C.; Quintini C., Ex situ liver machine perfusion: the impact of fresh frozen plasma, Liver Transplantation, 2, 2020.	30	2020	7.50
**48**	Webb A.N.; Lester E.L.W.; Shapiro A.M.J.; Eurich D.T.; Bigam D.L., Cost-utility analysis of normothermic machine perfusion compared to static cold storage in liver transplantation in the Canadian setting, American Journal of Transplantation, 2, 2022.	29	2022	14.50
**49**	Karangwa S.A.; Burlage L.C.; Adelmeijer J.; Karimian N.; Westerkamp A.C.; Matton A.P.; Van Rijn R.; Wiersema-Buist J.; Sutton M.E.; Op Den Dries S.; Lisman T.; Porte R.J., Activation of fibrinolysis, but not coagulation, during end-ischemic ex situ normothermic machine perfusion of human donor livers, Transplantation, 2, 2017.	27	2017	3.85
**50**	Weissenbacher A.; Bogensperger C.; Oberhuber R.; Meszaros A.; Gasteiger S.; Ulmer H.; Berchtold V.; Krendl F.J.; Fodor M.; Messner F.; Hautz T.; Otarashvili G.; Resch T.; Margreiter C.; Maglione M.; Irsara C.; Griesmacher A.; Raynaud M.; Breitkopf R.; Troppmair J.; Öfner D.; Cardini B.; Schneeberger S., Perfusate enzymes and platelets indicate early allograft dysfunction after transplantation of normothermically preserved livers, Transplantation, 4, 2022.	25	2022	12.50
**51**	Hann A.; Lembach H.; Nutu A.; Dassanayake B.; Tillakaratne S.; McKay S.C.; Boteon A.P.C.S.; Boteon Y.L.; Mergental H.; Murphy N.; Bangash M.N.; Neil D.A.H.; Issac J.L.; Javed N.; Faulkner T.; Bennet D.; Moore R.; Vasanth S.; Subash G.; Cuell J.; Rao R.; Cilliers H.; Russel S.; Haydon G.; Mutimer D.; Roberts K.J.; Mirza D.F.; Ferguson J.; Bartlett D.; Isaac J.R.; Rajoriya N.; Armstrong M.J.; Hartog H.; Perera M.T.P.R., Outcomes of normothermic machine perfusion of liver grafts in repeat liver transplantation (NAPLES initiative), British Journal of Surgery, 4, 2022.	24	2022	12.00
**52**	Mohkam K.; Nasralla D.; Mergental H.; Muller X.; Butler A.; Jassem W.; Imber C.; Monbaliu D.; Perera M.T.P.R.; Laing R.W.; García-Valdecasas J.C.; Paul A.; Dondero F.; Cauchy F.; Savier E.; Scatton O.; Robin F.; Sulpice L.; Bucur P.; Salamé E.; Pittau G.; Allard M.-A.; Pradat P.; Rossignol G.; Mabrut J.-Y.; Ploeg R.J.; Friend P.J.; Mirza D.F.; Lesurtel M., In situ normothermic regional perfusion versus ex situ normothermic machine perfusion in liver transplantation from donation after circulatory death, Liver Transplantation, 11, 2022.	24	2022	12.00
**53**	Karimian N.; Matton A.P.M.; Westerkamp A.C.; Burlage L.C.; Op Den Dries S.; Leuvenink H.G.D.; Lisman T.; Uygun K.; Markmann J.F.; Porte R.J., Ex situ normothermic machine perfusion of donor livers, Journal of Visualized Experiments, 99, 2015.	23	2015	2.55
**54**	Guo Z.; Zhao Q.; Jia Z.; Huang C.; Wang D.; Ju W.; Zhang J.; Yang L.; Huang S.; Chen M.; Zhu X.; Hu A.; Ma Y.; Wu L.; Chen Y.; Han M.; Tang Y.; Wang G.; Wang L.; Li L.; Xiong W.; Zhang Z.; Shen Y.; Tang Z.; Zhu C.; Chen X.; Hu X.; Guo Y.; Chen H.; Ma Y.; Zhang T.; Huang S.; Zeng P.; Lai S.; Wang T.; Chen Z.; Gong J.; Yu J.; Sun C.; Li C.; Tan H.; Liu Y.; Dong Y.; Sun C.; Liao B.; Ren J.; Zhou Z.; Andrea S.; Björn N.; Cai C.; Gong F.; Rong J.; Huang W.; Guan X.; Clavien P.A.; Stefan T.G.; Huang J.; He X., A randomized-controlled trial of ischemia-free liver transplantation for end-stage liver disease, Journal of Hepatology, 2, 2023.	23	2023	23.00
**55**	Hautz T.; Salcher S.; Fodor M.; Sturm G.; Ebner S.; Mair A.; Trebo M.; Untergasser G.; Sopper S.; Cardini B.; Martowicz A.; Hofmann J.; Daum S.; Kalb M.; Resch T.; Krendl F.; Weissenbacher A.; Otarashvili G.; Obrist P.; Zelger B.; Öfner D.; Trajanoski Z.; Troppmair J.; Oberhuber R.; Pircher A.; Wolf D.; Schneeberger S., Immune cell dynamics deconvoluted by single-cell RNA sequencing in normothermic machine perfusion of the liver, Nature Communications, 1, 2023.	23	2023	23.00
**56**	Boteon Y.L.; Laing R.W.; Schlegel A.; Wallace L.; Smith A.; Attard J.; Bhogal R.H.; Reynolds G.; PR Perera M.T.; Muiesan P.; Mirza D.F.; Mergental H.; Afford S.C., The impact on the bioenergetic status and oxidative-mediated tissue injury of a combined protocol of hypothermic and normothermic machine perfusion using an acellular haemoglobin-based oxygen carrier: The cold-to-warm machine perfusion of the liver, PLoS ONE, 10, 2019.	23	2019	4.60
**57**	Dondossola D.; Lonati C.; Zanella A.; Maggioni M.; Antonelli B.; Reggiani P.; Gatti S.; Rossi G., Preliminary experience with hypothermic oxygenated machine perfusion in an Italian liver transplant center, Transplantation Proceedings, 1, 2019.	22	2019	4.40
**58**	Lau N.-S.; Ly M.; Dennis C.; Liu K.; Kench J.; Crawford M.; Pulitano C., Long-term normothermic perfusion of human livers for longer than 12 days, Artificial Organs, 12, 2022.	22	2022	11.00
**59**	Webb A.N.; Izquierdo D.L.; Eurich D.T.; Shapiro A.M.J.; Bigam D.L., The actual operative costs of liver transplantation and normothermic machine perfusion in a Canadian setting, PharmacoEconomics—Open, 2, 2021.	20	2021	6.66
**60**	Pezzati D.; Ghinolfi D.; Balzano E.; De Simone P.; Coletti L.; Roffi N.; Rreka E.; Meacci L.; Campani D.; Mazzoni A.; Paolicchi A.; Caponi L.; Marchetti P.; Marselli L.; Filipponi F., Salvage of an octogenarian liver graft using normothermic perfusion: a case report, Transplantation Proceedings, 4, 2017.	20	2017	2.85
**61**	Patrono D.; De Carlis R.; Gambella A.; Farnesi F.; Podestà A.; Lauterio A.; Tandoi F.; De Carlis L.; Romagnoli R., Viability assessment and transplantation of fatty liver grafts using end-ischemic normothermic machine perfusion, Liver Transplantation, 5, 2023.	19	2023	19.00
**62**	Chapman W.C.; Barbas A.S.; D’Alessandro A.M.; Vianna R.; Kubal C.A.; Abt P.; Sonnenday C.; Barth R.; Alvarez-Casas J.; Yersiz H.; Eckhoff D.; Cannon R.; Genyk Y.; Sher L.; Singer A.; Feng S.; Roll G.; Cohen A.; Doyle M.B.; Sudan D.L.; Al-Adra D.; Khan A.; Subramanian V.; Abraham N.; Olthoff K.; Tekin A.; Berg L.; Coussios C.; Morris C.; Randle L.; Friend P.; Knechtle S.J., Normothermic machine perfusion of donor livers for transplantation in the united states: a randomized controlled trial, Annals of Surgery, 5, 2023.	18	2023	18.00
**63**	Huang V.; Karimian N.; Detelich D.; Raigani S.; Geerts S.; Beijert I.; Fontan F.M.; Aburawi M.M.; Ozer S.; Banik P.; Lin F.; Karabacak M.; Hafiz E.O.A.; Porte R.J.; Uygun K.; Markmann J.F.; Yeh H., Split-liver ex situ machine perfusion: a novel technique for studying organ preservation and therapeutic interventions, Journal of Clinical Medicine, 1, 2020.	17	2020	4.25
**64**	Matton A.P.M.; Selten J.W.; Roest H.P.; de Jonge J.; IJzermans J.N.M.; de Meijer V.E.; Porte R.J.; van der Laan L.J.W., Cell-free microRNAs as early predictors of graft viability during ex vivo normothermic machine perfusion of human donor livers, Clinical Transplantation, 3, 2020.	16	2020	4.00
**65**	Raigani S.; Karimian N.; Huang V.; Zhang A.M.; Beijert I.; Geerts S.; Nagpal S.; Hafiz E.O.A.; Fontan F.M.; Aburawi M.M.; Mahboub P.; Markmann J.F.; Porte R.J.; Uygun K.; Yarmush M.; Yeh H., Metabolic and lipidomic profiling of steatotic human livers during ex situ normothermic machine perfusion guides resuscitation strategies, PLoS ONE, 1, 2020.	15	2020	3.75
**66**	Zhang Z.; Tang Y.; Zhao Q.; Wang L.; Zhu C.; Ju W.; Wang D.; Yang L.; Wu L.; Chen M.; Huang S.; Gao N.; Zhu Z.; Zhang Y.; Sun C.; Xiong W.; Shen Y.; Ma Y.; Hu A.; Zhu X.; Rong J.; Cai C.; Guo Z.; He X., Association of perfusion characteristics and posttransplant liver function in ischemia-free liver transplantation, Liver Transplantation, 11, 2020.	15	2020	3.75
**67**	Thomas J.; Chen Q.; Roach A.; Wolfe S.; Osho A.A.; Sundaram V.; Wisel S.A.; Megna D.; Emerson D.; Czer L.; Esmailian F.; Chikwe J.; Kim I.; Catarino P., Donation after circulatory death heart procurement strategy impacts utilization and outcomes of concurrently procured abdominal organs, Journal of Heart and Lung Transplantation, 7, 2023.	14	2023	14.00
**68**	Mergental H.; Laing R.W.; Hodson J.; Boteon Y.L.; Attard J.A.; Walace L.L.; Neil D.A.H.; Barton D.; Schlegel A.; Muiesan P.; Abradelo M.; Isaac J.R.; Roberts K.; Perera M.T.P.R.; Afford S.C.; Mirza D.F., Introduction of the concept of diagnostic sensitivity and specificity of normothermic perfusion protocols to assess high-risk donor livers, Liver Transplantation, 5, 2022.	14	2022	7.00
**69**	De Carlis R.; Lauterio A.; Centonze L.; Buscemi V.; Schlegel A.; Muiesan P.; De Carlis L.; Carraro A.; Ghinolfi D.; De Simone P.; Ravaioli M.; Cescon M.; Dondossola D.; Bongini M.; Mazzaferro V.; Pagano D.; Gruttadauria S.; Gringeri E.; Cillo U.; Patrono D.; Romagnoli R.; Camagni S.; Colledan M.; Olivieri T.; Di Benedetto F.; Vennarecci G.; Baccarani U.; Lai Q.; Rossi M.; Manzia T.M.; Tisone G.; Vivarelli M.; Scalera I.; Lupo L.G.; Andorno E.; Meniconi R.L.; Ettorre G.M.; Avolio A.W.; Agnes S.; Pellegrino R.A.; Zamboni F., Current practice of normothermic regional perfusion and machine perfusion in donation after circulatory death liver transplants in Italy, Updates in Surgery, 2, 2022.	14	2022	7.00
**70**	Bral M.; Aboelnazar N.; Hatami S.; Thiesen A.; Bigam D.L.; Freed D.H.; Shapiro A.M.J., Clearance of transaminases during normothermic ex situ liver perfusion, PLoS ONE, 4, 2019.	14	2019	2.80
**71**	Pavel M.C.; Reyner E.; Fuster J.; Garcia-Valdecasas J.C., Liver transplantation from type II donation after cardiac death donor with normothermic regional perfusion and normothermic machine perfusion; [Trasplante hepático con injerto de donante en asistolia tipo 2 con perfusión regional normotérmica y máquina de perfusión normotérmica], Cirugia Espanola, 8, 2018.	14	2018	2.33
**72**	Meszaros A.T.; Hofmann J.; Buch M.L.; Cardini B.; Dunzendorfer-Matt T.; Nardin F.; Blumer M.J.; Fodor M.; Hermann M.; Zelger B.; Otarashvili G.; Schartner M.; Weissenbacher A.; Oberhuber R.; Resch T.; Troppmair J.; Öfner D.; Zoller H.; Tilg H.; Gnaiger E.; Hautz T.; Schneeberger S., Mitochondrial respiration during normothermic liver machine perfusion predicts clinical outcome, eBioMedicine, 2022.	12	2022	6.00
**73**	Del Turco S.; Cappello V.; Tapeinos C.; Moscardini A.; Sabatino L.; Battaglini M.; Melandro F.; Torri F.; Martinelli C.; Babboni S.; Silvestrini B.; Morganti R.; Gemmi M.; De Simone P.; Martins P.N.; Crocetti L.; Peris A.; Campani D.; Basta G.; Ciofani G.; Ghinolfi D., Cerium oxide nanoparticles administration during machine perfusion of discarded human livers: a pilot study, Liver Transplantation, 7, 2022.	12	2022	6.00
**74**	Lee A.C.H.; Edobor A.; Lysandrou M.; Mirle V.; Sadek A.; Johnston L.; Piech R.; Rose R.; Hart J.; Amundsen B.; Jendrisak M.; Millis J.M.; Donington J.; Madariaga M.L.; Barth R.N.; di Sabato D.; Shanmugarajah K.; Fung J., The effect of normothermic machine perfusion on the immune profile of donor liver, Frontiers in Immunology, 2022.	12	2022	6.00
**75**	Ciria R.; Ayllon-Teran M.D.; González-Rubio S.; Gómez-Luque I.; Ferrín G.; Moreno A.; Sánchez-Frías M.; Alconchel F.; Herrera C.; Martín V.; Sánchez-Hidalgo J.M.; Arjona-Sánchez Á.; Okuda Y.; Cabrera I.; Benavente B.; Rodriguez M.J.; Jurado-Martínez I.; Dueñas-Jurado J.M.; Robles-Arista J.C.; Rodriguez-Perálvarez M.; de La Mata García M.; López-Cillero P.; Briceño J., Rescue of discarded grafts for liver transplantation by ex vivo subnormothermic and normothermic oxygenated machine perfusion: first experience in Spain, Transplantation Proceedings, 1, 2019.	12	2019	2.40
**76**	Karimian N.; Raigani S.; Huang V.; Nagpal S.; Hafiz E.O.A.; Beijert I.; Mahboub P.; Porte R.J.; Uygun K.; Yarmush M.; Yeh H., Subnormothermic machine perfusion of steatotic livers results in increased energy charge at the cost of anti-oxidant capacity compared to normothermic perfusion, Metabolites, 11, 2019.	12	2019	2.40
**77**	Zhang Z.; Ju A.W.; Tang Y.; Wang L.; Zhu C.; Gao N.; Zhao Q.; Huang S.; Wang D.; Yang L.; Han M.; Xiong W.; Wu L.; Chen M.; Zhang Y.; Zhu Y.; Sun C.; Zhu X.; Guo Z.; He X., First preliminary experience with preservation of liver grafts from extended-criteria donors by normothermic machine perfusion in Asia, Annals of Transplantation, 2020.	12	2020	3.00
**78**	De Jong I.E.M.; Bodewes S.B.; Van Leeuwen O.B.; Oosterhuis D.; Lantinga V.A.; Thorne A.M.; Lascaris B.; Van Den Heuvel M.C.; Wells R.G.; Olinga P.; De Meijer V.E.; Porte R.J., Restoration of bile duct injury of donor livers during ex situ normothermic machine perfusion, Transplantation, 6, 2023.	11	2023	11.00
**79**	Schurink I.J.; de Haan J.E.; Willemse J.; Mueller M.; Doukas M.; Roest H.; de Goeij F.H.C.; Polak W.G.; Ijzermans J.N.M.; Dutkowski P.; van der Laan L.J.W.; de Jonge J., A proof of concept study on real-time LiMAx CYP1A2 liver function assessment of donor grafts during normothermic machine perfusion, Scientific Reports, 1, 2021.	11	2021	3.66
**80**	Liu Q.; Del Prete L.; Ali K.; Grady P.; Bilancini M.; Etterling J.; D’Amico G.; Diago Uso T.; Hashimoto K.; Aucejo F.; Fujiki M.; Eghtesad B.; Sasaki K.; Kwon C.H.D.; Chaudhry S.; Doi J.; Pita A.; New B.; Bennett A.; Cywinski J.; Miller C.; Quintini C., Sequential hypothermic and normothermic perfusion preservation and transplantation of expanded criteria donor livers, Surgery (United States), 3, 2023.	11	2023	11.00
**81**	Van Leeuwen O.B.; Fujiyoshi M.; Ubbink R.; Werner M.J.M.; Brüggenwirth I.M.A.; Porte R.J.; De Meijer V.E., Ex situ machine perfusion of human donor livers via the surgically reopened umbilical vein: a proof of concept, Transplantation, 10, 2019.	11	2019	2.20
**82**	Pavel M.-C.; Reyner E.; Molina V.; Garcia R.; Ruiz A.; Roque R.; Diaz A.; Fuster J.; Garcia-Valdecasas J.C., evolution under normothermic machine perfusion of type 2 DCD Livers discarded as nontransplantable, Journal of Surgical Research, 2019.	11	2019	2.20
**83**	Pérez Redondo M.; Alcántara Carmona S.; Fernández Simón I.; Villanueva Fernández H.; Ortega López A.; Pardo Rey C.; Duerto Álvarez J.; Lipperheide Vallhonrat I.; González Romero M.; Ballesteros Ortega D.; del Río Gallegos F.; Rubio Muñoz J.J., Implementation of a mobile team to provide normothermic regional perfusion in controlled donation after circulatory death: Pilot study and first results, Clinical Transplantation, 8, 2020.	10	2020	2.50
**84**	Haque O.; Raigani S.; Rosales I.; Carroll C.; Coe T.M.; Baptista S.; Yeh H.; Uygun K.; Delmonico F.L.; Markmann J.F., Thrombolytic therapy during ex-vivo normothermic machine perfusion of human livers reduces peribiliary vascular plexus injury, Frontiers in Surgery, 2021.	10	2021	3.33
**85**	Ohman A.; Raigani S.; Santiago J.C.; Heaney M.G.; Boylan J.M.; Parry N.; Carroll C.; Baptista S.G.; Uygun K.; Gruppuso P.A.; Sanders J.A.; Yeh H., Activation of autophagy during normothermic machine perfusion of discarded livers is associated with improved hepatocellular function, American Journal of Physiology—Gastrointestinal and Liver Physiology, 1, 2022.	10	2022	5.00
**86**	Huang C.; Huang S.; Tang Y.; Zhao Q.; Wang D.; Ju W.; Yang L.; Zhang J.; Wu L.; Chen M.; Zhang Z.; Zhu Z.; Wang L.; Zhu C.; Zhang Y.; Sun C.; Xiong W.; Shen Y.; Chen X.; Ma Y.; Hu A.; Zhu X.; Rong J.; Cai C.; Guo Z.; He X., Prospective, single-centre, randomised controlled trial to evaluate the efficacy and safety of ischaemia-free liver transplantation (IFLT) in the treatment of end-stage liver disease, BMJ open, 5, 2020.	10	2020	2.50
**87**	Lau N.-S.; Ly M.; Dennis C.; Ewenson K.; Ly H.; Huang J.L.; Cabanes-Creus M.; Chanda S.; Wang C.; Lisowski L.; Liu K.; Kench J.; McCaughan G.; Crawford M.; Pulitano C., Liver splitting during normothermic machine perfusion: a novel method to combine the advantages of both in-situ and ex-vivo techniques, HPB, 5, 2023.	9	2023	9.00
**88**	Sousa Da Silva R.X.; Bautista Borrego L.; Lenggenhager D.; Huwyler F.; Binz J.; Mancina L.; Breuer E.; Wernlé K.; Hefti M.; Mueller M.; Cunningham L.; De Oliveira M.L.; Petrowsky H.; Weber A.; Dutkowski P.; Hoffmann W.; Gupta A.; Tibbitt M.W.; Humar B.; Clavien P.A., Defatting of human livers during long-term e x situ normothermic perfusion: novel strategy to rescue discarded organs for transplantation, Annals of Surgery, 5, 2023.	9	2023	9.00
**89**	Zhao Q.; Huang S.; Wang D.; Zhang Z.; Wu L.; Yang L.; Ma Y.; Ji F.; Tang Y.; Wang L.; Zhu Z.; Zhu Y.; Xiong W.; Chen M.; Han M.; Zhou J.; Hu A.; Wang G.; Jiao X.; Zhu X.; Ju W.Q.; Guo Z.Y.; He X.S., Does ischemia free liver procurement under normothermic perfusion benefit the outcome of liver transplantation?, Annals of Transplantation, 2018.	9	2018	1.50
**90**	Seidita A.; Longo R.; Di Francesco F.; Tropea A.; Calamia S.; Panarello G.; Barbara M.; Gruttadauria S., The use of normothermic machine perfusion to rescue liver allografts from expanded criteria donors, Updates in Surgery, 1, 2022.	9	2022	4.50
**91**	Lau N.-S.; Ly M.; Dennis C.; Jacques A.; Cabanes-Creus M.; Toomath S.; Huang J.; Mestrovic N.; Yousif P.; Chanda S.; Wang C.; Lisowski L.; Liu K.; Kench J.G.; McCaughan G.; Crawford M.; Pulitano C., Long-term ex situ normothermic perfusion of human split livers for more than 1 week, Nature Communications, 1, 2023.	9	2023	9.00
**92**	Olumba F.C.; Zhou F.; Park Y.; Chapman W.C., Normothermic machine perfusion for declined livers: a strategy to rescue marginal livers for transplantation, Journal of the American College of Surgeons, 4, 2023.	9	2023	9.00
**93**	Chen Z.; Wang T.; Chen C.; Zhao Q.; Ma Y.; Guo Y.; Hong X.; Yu J.; Huang C.; Ju W.; Chen M.; He X., Transplantation of extended criteria donor livers following continuous normothermic machine perfusion without recooling, Transplantation, 6, 2022.	9	2022	4.50
**94**	Attard J.A.; Osei-Bordom D.-C.; Boteon Y.; Wallace L.; Ronca V.; Reynolds G.; Perera M.T.P.R.; Oo Y.H.; Mergental H.; Mirza D.F.; Afford S.C, Ex situ normothermic split liver machine perfusion: protocol for robust comparative controls in liver function assessment suitable for evaluation of novel therapeutic interventions in the pre-clinical setting, Frontiers in Surgery, 2021.	9	2021	3.00
**95**	Hefler J.; Leon-Izquierdo D.; Marfil-Garza B.A.; Meeberg G.; Verhoeff K.; Anderson B.; Dajani K.; Bigam D.L.; Shapiro A.M.J., Long-term outcomes after normothermic machine perfusion in liver transplantation—Experience at a single North American center, American Journal of Transplantation, 7, 2023.	8	2023	8.00
**96**	Ghinolfi D.; Melandro F.; Torri F.; Esposito M.; Bindi M.; Biancofiore G.; Basta G.; Del Turco S.; Lazzeri C.; Rotondo M.I.; Peris A.; De Simone P., The role of sequential normothermic regional perfusion and end-ischemic normothermic machine perfusion in liver transplantation from very extended uncontrolled donation after cardiocirculatory death, Artificial Organs, 2, 2023.	8	2023	8.00
**97**	Manzia TM, Toti L, Quaranta C, Blasi F, Tisone G. Liver transplantation with a normothermic machine preserved fatty nonagenarian liver: a case report. Int J Surg Case Rep, 2019.	8	2019	1.60
**98**	Raigani S, Santiago J, Ohman A, Heaney M, Baptista S, Coe TM, de Vries RJ, Rosales I, Shih A, Markmann JF, Gruppuso P, Uygun K, Sanders J, Yeh H. Pan-caspase inhibition during normothermic machine perfusion of discarded livers mitigates ex situ innate immune responses. Front Immunology, 2022.	8	2022	4.00
**99**	Allard MA, Castro-Benitez C, Imai K, Selten J, Lopez A, Sebagh M, Lemoine A, Sa Cunha A, Cherqui D, Castaing D, Vibert E, Adam R. Suitability of livers for transplantation when treated by normothermic machine perfusion. Clin Transplant, 6, 2018.	8	2018	1.33
**100**	Nasralla D, Lembach H, Mergental H, Mirza D, Friend P, Muiesan P, Perera M. Ex situ arterial reconstruction during normothermic perfusion of the liver. Transplant Direct, 9,2020.	7	2020	1.75

**Figure 2.**
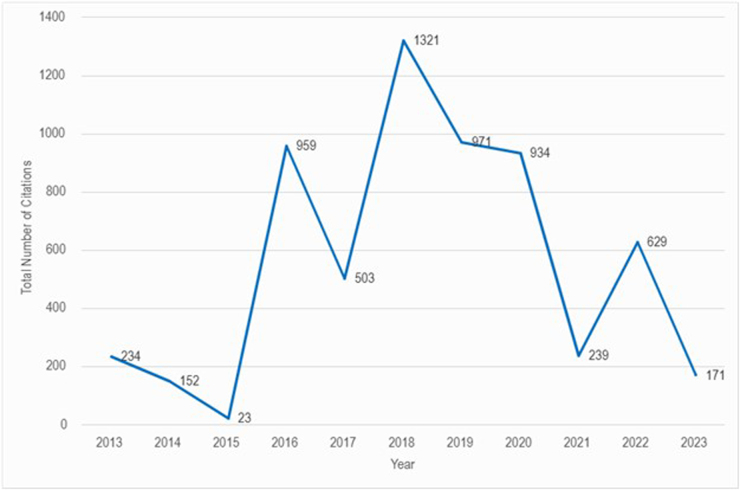
Total number of citations per year.

Figure 2 shows the trend of total citations, by year, of the 100 articles in the list. The number of citations rapidly rose from 2015, with the highest momentum observed in 2018. 2019 and 2020 had a similar citation trend, followed by a substantial dip in 2021. Another rise in the citation count was seen in 2022, with a subsequent dip in 2023.

### Year of publications, origins, and authorship

**Figure 3.**
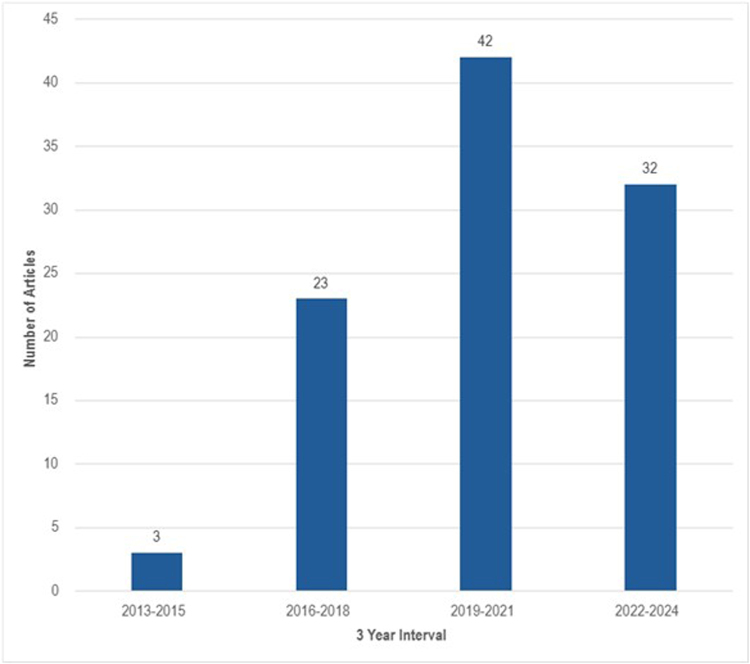
Number of articles in each 3-year interval.

**Figure 4.**
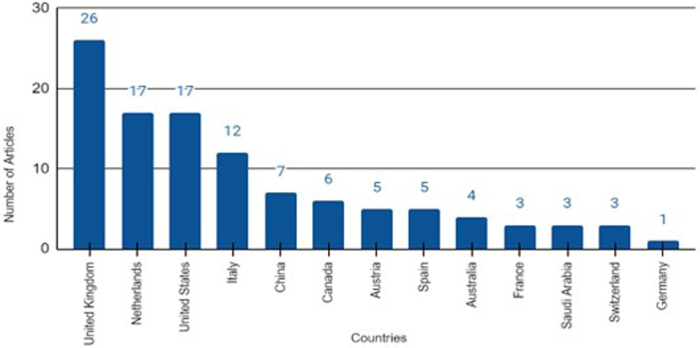
Number of articles originating from each country.

The 100 most cited articles were published between 2013 and 2023. During these 10 years, the highest number of articles (*n* = 42) were published in the 3 years from 2019 to 2021 (Fig. 3). The articles originated from 13 different countries, with 14% having authorship from more than one country. The top 3 countries of origin are the United Kingdom (*n* = 26), followed by the Netherlands (*n* = 17), and the United States (*n* = 17). (Fig. 4)

A broad range of 783 authors contributed to the top 100 articles. The number of authors per article ranged from 3 to 57, with a median of 14 (interquartile range of 7.75). Authors with eight or more articles in the list have been listed in Table 2. The most significant number of the most cited articles were co-authored by H. Mergental (*n* = 18), followed by R.J. Porte (*n* = 15), D.F. Mirza (*n* = 16), and P.J. Friend (*n* = 12). The authors’ H index, measured by author-specific citation frequency, has also been included to showcase the author’s overall impact on the world of science and research.

**Table 2:** Authors with eight or more articles in the top 100 list

Serial number	Author name	Number of articles	Author’s position (first)	Author’s position (last)	Author’s position (other)	Author affiliation	H-Index
1	Mergental H	18	4	1	13	Queen Elizabeth Hospital Birmingham, Birmingham, UK	38
2	Porte R.J.	17	0	12	5	Section of Hepatobiliary Surgery and Liver Transplantation, Department of Surgery, University of Groningen, University Medical Center Groningen, Groningen, The Netherlands.	87
3	Mirza D.F	16	0	5	11	Liver Unit, University Hospital Birmingham, Birmingham, UK.	86
4	Friend P.J	12	0	4	8	Nuffield Department of Surgical Sciences, University of Oxford, Oxford, UK.	78
5	Perera M.T.P.R.	11	1	1	9	Liver Unit, University Hospital Birmingham, Birmingham, UK.	39
6	Afford S.C	10	0	5	5	National Institute for Health Research (NIHR), Birmingham Biomedical Research Centre, University of Birmingham and University Hospitals Birmingham NHS Foundation Trust, Birmingham, United Kingdom;	39
7	Laing R.W	9	2	0	7	National Institute for Health Research (NIHR) Birmingham Liver Biomedical Research Unit and Centre for Liver Research, Institute of Immunology and Immunotherapy, Institute for Biomedical Research, College of Medical and Dental Sciences, University of Birmingham, Birmingham, UK.	20
8	Boteon Y.L	9	1	0	8	Liver Unit, Queen Elizabeth Hospital, University Hospitals Birmingham NHS Foundation Trust (UHBFT), Birmingham, UK.	22
9	Muiesan P	9	0	1	8	Liver Unit, Queen Elizabeth Hospital, University Hospitals Birmingham NHS Foundation Trust (UHBFT), Birmingham, UK.	72
10	Karimian N.	9	2		7	Section of Hepato-Pancreato-Biliary Surgery and Liver Transplantation, University of Groningen, University Medical Center Groningen; Surgical Research Laboratory, Department of Surgery, University of Groningen, University Medical Center Groningen	19
11	Coussios C.C	8	0	0	8	Institute of Biomedical Engineering, Department of Engineering Science, University of Oxford, Oxford, UK.	54
12	Schlegel A	8	0	0	8	Liver Unit, Queen Elizabeth Hospital, University Hospitals Birmingham NHS Foundation Trust (UHBFT), Birmingham, UK.	49
13	De Meijer V.E	8	0	1	7	Section of Hepatobiliary Surgery and Liver Transplantation, Department of Surgery, University Medical Center Groningen, University of Groningen, Groningen, the Netherlands.	41
14	Matton A.P.M	8	3	0	5	Section of Hepatobiliary Surgery and Liver Transplantation, Department of Surgery, University Medical Center Groningen, University of Groningen, Groningen, The Netherlands.	14
15	Wang L	8	1	0	7	Translational and Clinical Research Institute, Newcastle University, Newcastle upon Tyne, United Kingdom	7

**Figure 5.**
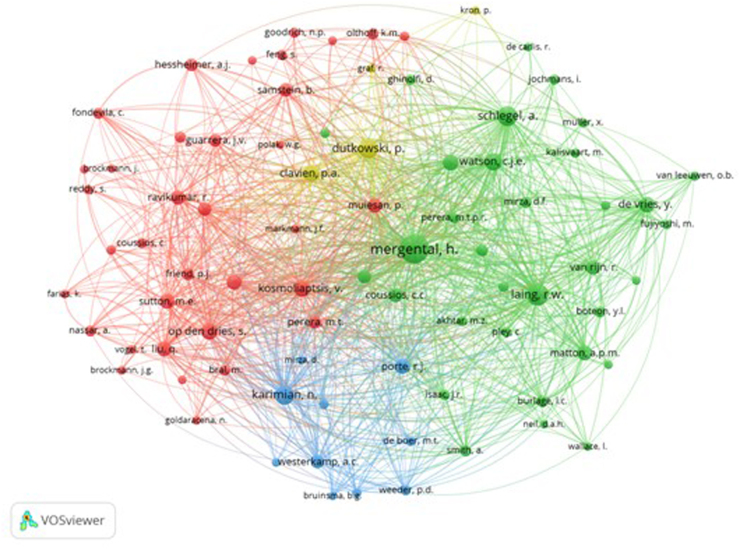
Co-author citation analysis.

Co-citation analysis encourages researchers to explore the intellectual structure of science and guide them about future developments. When authors are cited together in more papers, the relationship is stronger, and the co-citation strength is greater. Our co-citation analysis by authors involved 3983 authors, 82 of whom were cited at least 20 times. The authors who were most cited included Mergental H, followed by Laing R.W. and Schlegel A. Figure 5 depicts the analysis. The authors’ names are exhibited by a circle and a label.

### Journal and institutional affiliations

The 100 most cited articles were published in 19 different journals, with the top 4 producing nearly half of the articles in the list (47% of the top 100 most influential articles on normothermic machine perfusion). These journals are *Liver Transplantation* (*n* = 21), *Transplantation* (*n* = 10), *American Journal of Transplantation* (*n* = 10), and *Annals of Surgery* (*n* = 6). The impact factor of these 19 journals ranged from 1.6 to 4.7. There was no significant association between the impact factor of a journal and the number of articles published (*P*-value = 0.984). Journals having more than one article in the list of top 100 articles have been included in Table 3.

**Table 3:** Journals with two or more articles in the list

Rank	Journal name	Number of articles	Impact factor
1	Liver Transplantation	21	4.7
2	Transplantation	10	5.3
3	American Journal of Transplantation	10	8.9
4	Annals of Surgery	6	7.5
5	PLoS ONE	4	2.9
6	Transplantation Direct	3	1.9
7	Transplantation Proceedings	3	0.8
8	Nature communications	3	14.7
9	BMJ Open	3	2.4
10	Frontiers in Immunology	3	5.7
11	Clinical Transplantation	3	1.9
12	Nature Biotechnology	2	33.1
13	British Journal of Surgery	2	8.6
14	Journal of Hepatology	2	26.8
15	HPB	2	2.7
16	Surgery (United States)	2	3.2
17	Artificial Organs	2	2.2
18	Updates in Surgery	2	2.4
19	Annals of Transplantation	2	1.1
20	Frontiers in Surgery	2	1.6

Different institutions are affiliated with our list of the top 100 articles. University Hospitals Birmingham NHS Foundation Trust led with 19 publications, followed by University Medical Center Groningen with 16 publications and Massachusetts General Hospital with 9 publications. Table 4 lists the institutions having 5 or more articles in the list.

**Table 4:** Institutions affiliated with five or more articles in the list

Institutions affiliated with 5 or more articles in the list	
Institute	Number of documents
University Hospitals Birmingham NHS Foundation Trust	19
University Medical Center Groningen	16
Massachusetts General Hospital	9
University Hospital Zurich	7
Sun Yat-sen University	7
University of Alberta	6
Cleveland Clinic	5
University of Oxford	5

### Topical distribution and type of article

Table 5 shows the different categories the 100 most cited articles can be classified into according to the aims of the study: normothermic machine perfusion in discarded livers and extended criteria donors (*n* = 15), comparison of perfusion methods (*n* = 19), parameters and biomarkers to assess viability and outcomes (*n* = 30), sequential use of NMPs with other perfusion methods (*n* = 10), administration of agents or treatment during NMP (*n* = 9), and extended NMP perfusion (*n* = 6). Other articles aimed at developing protocols, analyzing cost, and practicing split liver perfusion techniques.

**Table 5:** Article description

Description		Frequency
Primary Aim (*n* = 100)	Use of NMP in discarded livers and ECDs	15
	Comparison of perfusion methods	19
	Sequential use of NMP with other perfusion methods	10
	Parameters and biomarkers to assess viability and outcomes	30
	Concurrent administration of agents/treatment during NMP	9
	Extended NMP perfusion	6
	Other	11

### Funding, conflict of interest, race, and gender

More than two-thirds of the articles received funding (*n* = 78). It was found that the presence of funding had no significant effect on the number of citations (*P* = 0.987) Government institutions and agencies sponsored more than half of the funded articles (*n* = 58), out of which the Medical Research Council was the biggest source (*n* = 8). Among the private funders, Wellcome Trust sponsored the most articles (*n* = 9). Furthermore, most articles received institutional support from their respective affiliation. Table 6 highlights the different sources of funding. Over a third (*n* = 35) of articles reported a conflict of interest. However, our analysis reported no significant association between conflict of interest and number of citations of the article (*P* = 0.856)

**Table 6:** Type of funding with the total number of articles

Type of funding	Number of articles
Private	20
Government	30
Funded by both institutions	28
Not funded	22

**Figure 6.**
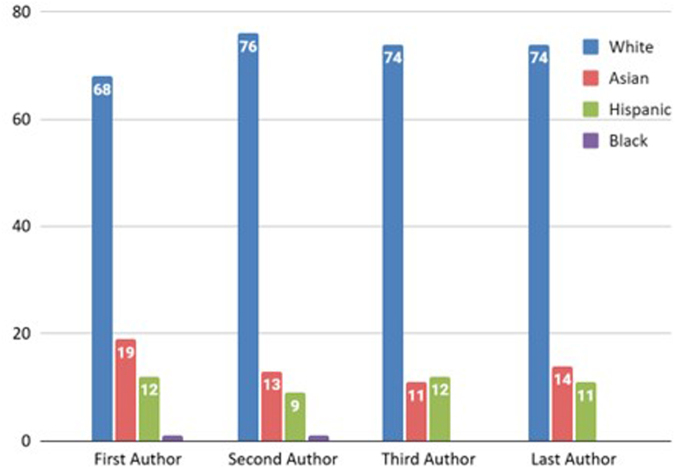
Racial distribution of the first, second, third, and last authors in the top 100 cited articles.

The overwhelming majority of the primary authors were Caucasian (*n* = 68), followed by Asian (*n* = 19) and Hispanic (*n* = 12). Only a single Black was the primary author. A similar trend was seen among the senior authors, with the majority being Caucasian (*n* = 75), followed by Asians (*n* = 14) and Hispanics (*n* = 11). No Black author held senior authorship. Figure 6 depicts a visual representation of this analysis.

**Figure 7.**
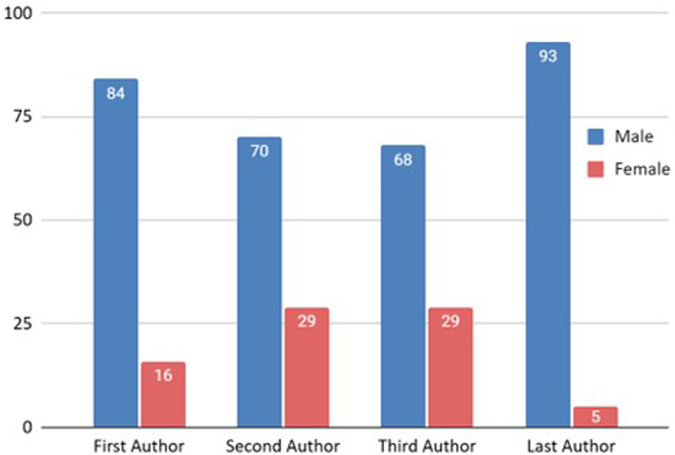
Gender distribution of the first, second, third, and last authors in the top 100 cited articles.

A large number of the primary authors were males (*n* = 86). An identical trend was observed in the senior authorship position, primarily held by males (*n* = 95). We found no significant association between the gender of the first and senior authors (*P* = 0.274) or between the gender of the first author and the number of citations of the article (*P* = 0.781). Figure 7 displays the variation of gender among the first, second, third, and last authors.

### Review articles

Table 7 shows the top ten most cited review articles regarding normothermic liver perfusion, along with their total citations and citations per year.

**Table 7:** Top 10 review articles

Rank	Article	Total citations	Average citations per year
1	Morrissey PE, Monaco AP. Donation after circulatory death. Transplantation Journal. 2014;97(3):258–64.	212	21.2
2	Maathuis MHJ, Leuvenink HGD, Ploeg RJ. Perspectives in organ preservation. Transplantation. 2007;83(10):1289–98.	182	10.7
3	Dutkowski P, Olivier de Rougemont, Pierre-Alain Clavien. Machine perfusion for “marginal” liver grafts. American Journal of Transplantation. 2008;8(5):917–24.	104	6.5
4	Jochmans I, M. Jaleel Akhtar, Nasralla D, Peri Kocabayoglu, Boffa C, M Kaisar, *et al.* Past, present, and future of dynamic kidney and liver preservation and resuscitation. 2016;16(9):2545–55.	101	12.6
5	Watson CJ, Jochmans I. From “gut feeling” to objectivity: machine preservation of the liver as a tool to assess organ viability. Current Transplantation Reports. 2018;5(1):72–81.	101	16.8
6	Graham JP, Guarrera JV. “Resuscitation” of marginal liver allografts for transplantation with machine perfusion technology. 2014;61(2):418–31.	86	8.6
7	Weissenbacher, A., Vrakas, G., Nasralla, D. and Ceresa, C.D.L. The future of organ perfusion and re-conditioning. Transpl Int. 2019;32: 586–597.	86	17.2
8	Monbaliu D, Brassil J. Machine perfusion of the liver: past, present and future. Current Opinion in Organ Transplantation. 2010;15(2):160–6.	84	6.0
9	Ravikumar R, Henri, Friend PJ. Normothermic liver preservation: a new paradigm? Transplant International. 2015 Apr;28(6):690–9.	82	9.1
10	Resch T, Cardini B, Oberhuber R, Weissenbacher A, Dumfarth J, Krapf C, Boesmueller C, Oefner D, Grimm M, Schneeberger S. Transplanting Marginal Organs in the Era of Modern Machine Perfusion and Advanced Organ Monitoring. Front Immunol. 2020 May 12;11:631.	82	20.5

## Discussion

### Research activity

Despite existing analyses of top-cited articles in liver transplantation, normothermic machine perfusion (NMP) still needs to be explored.^[16,17]^ Therefore, this study aims to analyze the characteristics of the top 100 most cited articles on normothermic machine perfusion, thereby addressing a significant gap in the existing literature. Table 1 highlights the 100 most-cited publications on normothermic machine perfusion, identified through comprehensive bibliometric analysis, providing the foundation for our modern understanding of liver transplantation. The citation activities and distribution of these influential studies offer valuable insights into its evolution, complementing advancements in organ preservation techniques for liver transplantation.

The current progression of research on normothermic machine perfusion of human livers can be attributed to the success of machine perfusion on animal livers. The earliest documented experimentation was in the late 1960s and 1970s, mostly done on porcine livers, rabbits, and rats, where hypothermic and normothermic conditions were explored for liver preservation.^[18-20]^ Successful experimental applications of NMP on livers were conducted in the early 2000s, and notable progress has been made since. The superiority of normothermic machine perfusion over static cold storage has been exhibited in various research in the past two decades, with another advantage of the ability to perform viability assessments of donor liver grafts before transplantation.^[21-23]^

The first clinical trial of normothermic machine perfusion in human livers was initiated in 2013 on 20 donor livers^[24]^, which led to a new beginning in preserving human livers. Figure 2 shows that most articles (*n* = 97) among the 100 top-cited were published between 2015 and 2023, reflecting the field’s ongoing progress and modern approach. Other rapidly evolving specialties, such as neurosurgery, show analogous temporal patterns in publication.^[3]^ Figure 3 reveals that the peak research activity in normothermic machine perfusion occurred between 2016 and 2020, with the top 100 articles receiving the most citations during this period and a subsequent decline in the citations, suggesting a passing peak investigation period.

Since then, researchers have discovered a deeper understanding of NMP, identifying better indicators for biochemical markers, and experimented with sequential dual or triple perfusion methods.^[25-27]^ For a change in the universal preservation of static cold storage to other machine perfusion methods, a holistic analysis is crucial to identify graft-tailored preservation with a discussion between regulatory authorities and the transplantation community needed to facilitate research with intense urgency.^[28]^

### Journals

Table 3 lists the journals publishing the most influential research papers, which, according to Bradford’s Law, are “core journals” which states that core journals are those that are most frequently cited in the literature of a particular field, and so they are likely to be of value to researchers within that discipline.^[29]^ Around half of the top 100 most cited articles on normothermic machine perfusion appeared in the following journals: Liver Transplantation, Transplantation, American Journal of Transplantation, and Annals of Surgery. Although journal impact factors and citation counts are not meant to be directly compared, there is still divergence regardless of the expectations. Moreover, the journal with the highest impact factor (26.8) received only two citations, and the journal with the lowest impact factor (0.8) in the top 100 most cited articles received three citations. The journal’s standing does not purely determine the allocation of the citations.^[30]^ Additionally, the difference in calculation periods can significantly impact how journal performance is evaluated, especially across disciplines with varying citation patterns.^[31]^

### Content

The progression of research in normothermic machine perfusion can be attributed to its significant results from experimentation on non-human subjects, mainly porcine mammals, rodents, and rabbits in the early 2000s. Research into normothermic machine perfusion in the human liver progressed further in the last decade, with expectations of further improvements. Our top 100 most cited list highlights the evolving applications and optimization of normothermic machine perfusion (NMP) in liver transplantation. By examining the 100 most-cited publications, we identified key findings in Table 5, which underscores the need for standardized protocols, predictive models, and long-term outcome studies. Generally, this analysis produces a significant role NMP continues to assume in liver transplantation and generates possibilities in graft function and organ availability with an even broader prospective application in other settings of organ transplantation in the future. A notable number of articles on the list compared the various perfusion methods against the standard cold storage method. Dual or triple perfusion techniques with hypothermic or oxygenated and sub-normothermic perfusion were compared and contrasted against the standard procedure. Furthermore, multiple biomarkers were analyzed to evaluate clinical viability, outcomes, and safety associated with NMP and thus creating reference clinical criteria for transplantation using NMP.

### Origins and institutions

The geographic distribution of the most-cited papers reflects much diversity in the landscape of research, among which top cited papers originate primarily from the United Kingdom, closely followed by the Netherlands and the USA. European countries are most prominent in all the tiers, with Italy, Austria, Spain, and France also making significant contributions to other nations mentioned in Figure 4. Meanwhile, China being the only contributor from Asia is also noted. Africa (*n* = 0) and South America (*n* = 1) contributed the least. These trends highlight the excellence of European research and collaboration between various countries while further identifying gaps in global research. The articles in our study were affiliated with a multitude of institutions. Table 4 lists the most contributing institutions, of which the top two prolific institutions in terms of publications were University Hospitals Birmingham NHS Foundation Trust and University Medical Centre Groningen, publishing 19 and 16 articles, respectively. These institutions demonstrate exceptional leadership in NMP research, supplemented by significant contributions from other universities mentioned in the figure.

### Gender of first and senior authors

Figure 7 demonstrates a substantial gender disparity in our literature on normothermic machine perfusion. Our analysis revealed that 85% of first authors were male, while only 15% were female. Furthermore, senior authorship positions showed significant gender disparity, with 95% males and 5% females holding the positions. Unfortunately, the disparities in gender among the first and last authors are also noted in academic surgery. A recent study revealed a percentage of 23.8% and 14.7% of women among the first and last authors, respectively^[32]^. Our figures show less significant numbers of female authorship. Another study also noted that while women’s first authorship was higher in fields like public health, infectious diseases, and kidney transplantation, there were lower numbers in surgical innovations, organ preservation, living donor transplantation, liver and lung transplantation, and multiorgan transplantation^[6]^. This discrepancy raises concerns regarding the representation and prospects of female researchers. While interpreting the results, it is imperative to consider the citation metrics and career stage, as these factors may contribute to the underrepresentation of females in early career stages. Our results imply no direct correlation between senior author gender and citations. A grassroots strategy is necessary to address the gender gap in academic writing. Countries and institutions must adopt regional and national strategies to foster career advancement, professional development, and research opportunities for women in surgical, anesthesia, and other fields^[33]^. Furthermore, ensuring mentorship and representation is crucial in narrowing gender gaps and promoting equitable growth in women. We must learn from the Johns Hopkins School of Medicine Equity Issues Task Force methodology, which operated from 1990 to 1995^[34]^. This task force identified and enhanced the representation of women, resulting in a 550% increase in women-associated professors. More exploration into the factors limiting women’s engagement in surgical research and assessing potential strategies to enhance their leadership in surgical publications is necessary to narrow the wide gender disparity further.

### Funding and conflict of interest

An overwhelming majority of the articles were funded. Previous studies have shown that funding tends to result in higher citation counts.^[35]^ No significance was established between our study’s presence or type of funding and citation count. Interestingly, most of the articles funded in our study reported positive results, and the majority came from influential organizations. Wellcome Trust had the first funding source, followed by the Medical Research Council UK and other institutes and universities. Such a funding landscape carries essential implications regarding the connection of these stakeholders and the priorities and results of the research. However, many articles mentioned that sponsors were blind to the study, except for providing funds and machine perfusion devices. This can be understandable as there are limited brands of machine perfusion devices, and the cost of purchasing or leasing the device and the staff costs of running it need to be considered.^[36]^

Furthermore, our study revealed that more than a third of the articles reported a conflict of interest. Although our analysis showed no significant association between conflict of interest and article citation, such conflicts can skew the results.

### Limitations

With all the best endeavors to make it bias-free, some limitations inherent in bibliometric analysis remain. Studies have shown that although Scopus indexes a more extensive journal set, which is helpful for both keyword searching and citation analysis, it is limited to the articles published after 1995, resulting in the omission of those articles.^[14]^ In a related matter, there has been some discussion about whether self-citations are skewing the results of such analyses, mainly when a handful of high-profile authors contribute a substantial number of highly cited works. By artificially boosting the h-index, self-citations can shape research direction and influence perceived author impact by introducing citation bias. An updated h-index that accounts for and excludes self-citations can provide a more accurate measure of academic impact.^[37]^ Implementing guidelines to keep self-citation rates below 10% would help maintain fair competition and enhance academic credibility.^[38]^ However, another analysis found that self-citations have minimal effect on bibliometric metrics. Our study found a self-citation rate of 18%, far above what is generally reported for the discipline of general medicine (6.5%)^[39,40]^ and in biomedical journals as a whole, which was 12%, according to an article.^[41]^ This difference makes it a matter of interest to understand how such biases and resultant effects might influence the outcome of such analyses.

Additionally, this analysis relies only on Scopus as the regular reference source and thus may not include articles not indexed or referred to by the database. Searching multiple databases was impractical due to differences in indexing and citation metrics, which can result in slight variations in results. Potential shortcomings in the database may have led to the omission of articles published before 1980, as Scopus is known to sometimes miss older citations.^[42]^ Additionally, recently published articles may not have accumulated enough citations to be included in the list, potentially overlooking emerging landmark research.

### Review articles

This study is supplemented by comprehensive review articles integrating the evolving landscape in Normothermic Machine Perfusion in liver transplantation. Table 6 lists the top 10 most frequently cited review articles in this field, providing clinicians and researchers with a resource that benefits their understanding of the current cutting-edge advancements and evidence-based practices in NMP.

## Conclusion

Research on normothermic machine perfusion is rapidly growing and encompasses a variety of countries and institutions. Our article comprises a list of highly cited articles that have been particularly analyzed from medical literature and can aid research in the field and healthcare professionals. Furthermore, our research shows that normothermic machine perfusion is an evolving research topic not only in the fields of clinical medicine but also in biochemistry, bioengineering, molecular biology, and more. Collaborations between prominent researchers within the field and authors from different countries were identified in creating truly impactful studies.

## Data Availability

All data generated or analyzed during this study are included in this article. Further inquiries can be directed to the corresponding author.
